# Targeting CDK4/6 in Combination with Phage-Based Anti-HER2 Vaccination Overcomes Immune Evasion and Enhances the Anticancer Response in Breast Cancer

**DOI:** 10.3390/pharmaceutics18070881

**Published:** 2026-07-18

**Authors:** Junbiao Wang, Alessia Lamolinara, Daniele Tomassoni, Laura Conti, Chiara Cossu, Antonino Di Lorenzo, Mara Giangrossi, Daniela Lufrano, Varshini Vaithianathan, Fiorenza Orlando, Fabiola Olivieri, Serena Marcozzi, Daniela Beghelli, Barbara Belletti, Augusto Amici, Maurizio Falconi, Federica Cavallo, Manuela Iezzi, Cristina Marchini

**Affiliations:** 1 School of Biosciences and Veterinary Medicine, University of Camerino, Via Gentile III da Varano, 62032 Camerino, Italy; junbiao.wang@unicam.it (J.W.); daniele.tomassoni@unicam.it (D.T.); mara.giangrossi@unicam.it (M.G.); daniela.lufrano@unicam.it (D.L.); varshi.vaithianathan@studenti.unicam.it (V.V.); daniela.beghelli@unicam.it (D.B.); augusto.amici@unicam.it (A.A.); 2 Center for Advanced Studies and Technology, Department of Neurosciences, Imaging and Clinical Sciences, G. d’Annunzio University of Chieti-Pescara, 66013 Chieti, Italy; alessia.lamolinara@unich.it (A.L.); miezzi@unich.it (M.I.); 3 Department of Molecular Biotechnology and Health Sciences, Molecular Biotechnology Center “Guido Tarone”, University of Torino, 10126 Torino, Italy; laura.conti@unito.it (L.C.); chiara.cossu@unito.it (C.C.); antonino.dilorenzo@unito.it (A.D.L.); federica.cavallo@unito.it (F.C.); 4 Department of Biological Sciences, Faculty of Exact Sciences, National University of La Plata, CONICET (Consejo Nacional de Investigaciones Científicas y Técnicas), 47 & 115, La Plata B1900AVW, Argentina; 5 Experimental Animal Models for Aging Unit, Scientific Technological Area, IRCCS INRCA, 60100 Ancona, Italy; F.ORLANDO@inrca.it; 6 Department of Clinical and Molecular Sciences, Università Politecnica Delle Marche, 60100 Ancona, Italy; f.olivieri@staff.univpm.it; 7 Biogerontology Center and Geriatric Mouse Clinic, IRCCS INRCA, 60121 Ancona, Italy; s.marcozzi@inrca.it; 8 Molecular Oncology Unit, Centro di Riferimento Oncologico di Aviano (CRO Aviano), IRCCS, National Cancer Institute, 33081 Aviano, Italy; bbelletti@cro.it

**Keywords:** bacteriophages, breast cancer, cancer vaccines, HER2, combination therapy, CDK4/6 inhibitors, palbociclib

## Abstract

**Background/Objectives**: Cancer vaccines represent the next frontier in immunotherapy, aiming to elicit long-lasting protective anti-tumor immune responses. Human epidermal growth factor receptor 2 (HER2) is a well-established therapeutic target in breast cancer. Active immunization with HER2-displaying M13 bacteriophages can induce a therapeutic immune response against HER2-positive breast cancer, offering a promising alternative to trastuzumab. However, the duration of anticancer immune protection triggered by anti-HER2 phage-based vaccines is limited by tumor-immune suppressive mechanisms. **Methods**: In this study, two vaccination cycles with ECTM phages displaying the extracellular (EC) and transmembrane (TM) domains of human HER2 were combined with palbociclib, a CDK4/6 inhibitor, to enhance antitumor immunity in the clinically relevant Δ16HER2 transgenic preclinical model of breast cancer. **Results**: The proposed combination treatment resulted in a better and long-lasting control of tumor growth rate and multiplicity than either palbociclib or phage vaccination alone, correlating with a significantly stronger anti-HER2 humoral response (IgG2a isotype). Analysis of the tumor immune infiltrate revealed an increased presence of CD8^+^ T cells concomitant with a reduction in FoxP3^+^ regulatory T cells (Tregs) in tumors explanted from mice receiving the combination therapy. **Conclusions**: These preclinical results provide a rationale for the clinical translation of CDK4/6 inhibitors combined with anti-HER2 active immunotherapies in breast cancer, as they may yield sustained antitumor responses by reverting the immunosuppressive tumor environment.

## 1. Introduction

Global breast cancer incidence and associated mortality are projected to rise by 2050, underscoring the urgent need for novel and more effective therapeutic strategies [1]. The human epidermal growth factor receptor 2 (HER2) is overexpressed in 20% of breast cancers, and it is associated with poor prognosis, although HER2-targeted therapies dramatically improved survival outcomes [2]. Indeed, the effectiveness of anti-HER2 targeted drugs, including the monoclonal antibody trastuzumab, is often compromised by drug resistance mechanisms [3]. Moreover, trastuzumab therapy is associated with cardiotoxicity [4], which increases significantly with age [5]. Anti-HER2 vaccination represents a promising alternative to therapeutic monoclonal antibodies, being potentially able to induce durable immunologic and clinical responses. However, cancer vaccine efficacy is limited by tumor immune suppressive mechanisms, and currently no anti-HER2 cancer vaccine has been approved yet for human use [6]. Among cancer vaccine platforms, phage-displayed vaccines hold considerable potential, combining the intrinsic immunogenicity of viral particles with the high specificity of phage-displayed antigens [7,8,9]. In particular, M13 filamentous bacteriophages represent reliable immunogen carriers, as they are nonpathogenic and nonlytic viruses that infect and replicate exclusively in *Escherichia coli* cells harboring an F′ episome. Phages are internalized and processed by antigen-presenting cells, thereby inducing both B cell- and T cell-mediated immune responses, and they are immunogenic even in the absence of adjuvants [10]. Previous reports on anti-MAGE (Melanoma Antigen Gene) vaccination indicate that engineered filamentous bacteriophage virions can increase the immunogenicity of delivered tumor-associated antigens [11]. Phage vaccination was also shown to be safe and effective in evoking tumor-specific immune responses in malignant melanoma animal models [12,13] and multiple myeloma patients [14]. Consistently, we reported that vaccines based on filamentous bacteriophage M13 virions, engineered to display on their surface antigenic portions of HER2, can overcome tolerance against the HER2 self-antigen and elicit a protective immune response. Indeed, they were able to prevent breast cancer development in Δ16HER2 mice, which spontaneously develop HER2-positive and estrogen receptor-positive mammary carcinomas [15,16] and exhibit immunological tolerance to the human HER2 antigen [17]. However, when tested in therapeutic settings, the duration of anticancer immune protection triggered by these phage-based vaccines was limited by tumor immune evasion mechanisms [18]. Alterations of the cell cycle are present in virtually all human cancers. Cyclin E-cyclin-dependent kinase 2 (CDK2) and cyclin D-CDK4/6 complexes are crucial drivers of the cell cycle, as they promote the G1-to-S transition through phosphorylation of the retinoblastoma protein (pRb). Pharmacologic inhibitors of CDK4/6, by restoring the cell-cycle control, have shown significant activity against several tumors [19]. Recently, some CDK4/6 inhibitors, including palbociclib and abemaciclib, have been approved by the Food and Drug Administration (FDA) and the European Medicines Agency (EMA) for the treatment of hormone receptor (HR)-positive, HER2-negative breast cancer in combination with endocrine therapy, and are under clinical evaluation in other breast cancer subtypes [20]. In HER2-positive breast cancer, a combination of CDK4/6 inhibitors with HER2-targeted therapies showed promise in preclinical studies, and it is currently being tested in multiple clinical trials [21,22]. Of note, beyond inducing tumor cell cycle arrest, CDK4/6 inhibitors also promote antitumor immunity by increasing the functional capacity of tumor cells to present antigen and reducing the immunosuppressive regulatory T cells (Treg) population through the selective suppression of their proliferation [23]. Treg cells are a CD4^+^ T-cell subset essential for maintaining immune tolerance. In the tumor microenvironment, Treg cells contribute to tumor immune evasion and their downregulation by CDK4/6 inhibitors was demonstrated in both preclinical models and breast cancer patients [24,25]. Moreover, CDK4/6 inhibition skews newly activated CD8^+^T cells toward a memory phenotype as reported by Heckler et al. [25]. Thus, treatment with CDK4/6 inhibitors can re-educate the anti-cancer immune response in breast cancer patients by downregulating immunosuppressive Treg cells and activating effector T cells [24]. In this study, we demonstrate that combining a phage-based anti-HER2 vaccine with the CDK4/6 inhibitor palbociclib significantly improves the therapeutic control of HR^+^/HER2^+^ breast cancer in Δ16HER2 transgenic mice compared with either monotherapy. The combination treatment resulted in more durable tumor growth suppression and reduced tumor multiplicity, which were associated with enhanced anti-HER2 humoral immunity, HER2 downregulation, and a profound reshaping of the tumor immune microenvironment. These findings support the concept that CDK4/6 inhibition can enhance the efficacy of active anti-HER2 immunization by simultaneously overcoming immune tolerance to HER2 and mitigating tumor-driven immunosuppression. Another aspect that deserves to be clarified is the impact of phage-based vaccines on gut microbioma. Although it has been recently reported that a phage-based vaccine against SARS-CoV-2 did not affect the gut microbiota [26], phages can potentially manipulate it. This is a double-edged sword, because intestinal microbiota can influence vaccine responses [27] and enhance patient responses to immunotherapy [28]. In this study, the ECTM-phage vaccine did not affect overall the relative abundance of most bacterial families. However, a selective increase in *Lachnospiracea*, positively correlated with a stronger therapeutic response to trastuzumab [29], was observed in vaccinated mice.

## 2. Materials and Methods

### 2.1. Phage Production and Purification

Engineered M13 bacteriophages, displaying on their surface the EC and TM domains of human HER2 (ECTM phages) in fusion with the coat protein pIII, were produced as previously described [17,18,30]. ECTM phages were released by TG1 bacterial cells, after transformation with recombinant phagemid vector pIF6 followed by isopropyl β-d-1-thiogalactopyranoside (IPTG) induction and super-infection with M13K07 helper phages. In brief, *Escherichia coli* TG1 cells were transformed with recombinant pIF6-phagemid, grown overnight in 2× TY medium with 100 µg/mL ampicillin and 1% glucose at 37 °C, and then, when they reached the log phase of growth (optical density (O.D.) corresponding to 0.4–0.5 at 600 nm), they were superinfected with the helper phage M13K07 at a multiplicity of infection (M.O.I.) of 20:1, incubated for 30 min at 37 °C without shaking, followed by 30 min at 37 °C with shaking. TG1 cells were then centrifuged for 10 min at 2465× *g*, and the cell pellet was resuspended in 2× TY medium supplemented with 100 µg/mL ampicillin, 70 µg/mL kanamycin and 200 µmol/L of IPTG and grown overnight at 30 °C. Polyethylene glycol (PEG)-based precipitation was used to purify M13 recombinant phages, which were harvested from the supernatant after centrifugation of the infected culture for 30 min at 352× *g*. In detail, phages were precipitated with a 3:10 *v*/*v* ratio of PEG-NaCl (20% PEG, 2.5 M NaCl) through incubation for 2 h on ice, pelleted by centrifugation at 4 °C for 2 h at 3220× *g* and suspended in 2 mL of PBS. They were subsequently filtered through a 0.22 µm Millipore filter (Merck, Darmstadt, Germania) and then quantified by colony-forming assay before being used as anti-HER2 vaccines in ∆16HER2 transgenic mice. The concentration of virion particles was verified by UV absorption spectrometry using the formula: virions/mL = [(A269 − A320) × 16 × 10^16^]/(number of bases/virion).

### 2.2. Mice Immunization and Palbociclib Treatment

Δ16HER2 transgenic mice (FVB/NHsd-Tg(D16HER2-LUC)6157Acam) [15] were housed under controlled temperature (20 °C) and circadian cycle (12 h light/12 h dark) in the animal facility of the University of Camerino. The animals were fed a chow diet (Mucedola s.r.l., Milan, Italy) and tap water ad libitum. Δ16HER2 transgenic mice were treated in accordance with the U.K. Animals (Scientific Procedures) Act, 1986 and associated guidelines, EU Directive 2010/63/EU for animal experiments and with the 3Rs principles. All animal experiments were authorized by the Italian Ministry of Health (#773/2023-PR) and by the Animal Research Committee of the University of Camerino (OPBA). For mouse colony genotyping, DNA was extracted from tail biopsies, and it was routinely used for genotyping by PCR. The following primers (Sigma) were used: Δ16HER2 For: 5′-GGTCTGGACGTGCCAGTGTGA-3′; Δ16HER2 Rev: 5′-GATAGAATGGCGCCGGGCCTT-3′.

Δ16HER2 transgenic female mice were vaccinated by intraperitoneal (i.p.) injection of ECTM-phages (1 × 10^11^ CFU/mouse in 0.1 mL PBS) at 12, 14, and 16 weeks of age (first vaccination cycle), and at 20, 22, and 24 weeks of age (second vaccination cycle). Empty phages were used as a control. Palbociclib (100 mg/Kg) (PD-0332991 hydrochloride (CliniSciences, Guidonia Montecelio, Italy) was diluted in Ringer’s lactate solution and administered to Δ16HER2 mice by oral gavage with plastic feeding tubes for 5 days a week, from 15 to 24 weeks of age, alone or in combination with anti-HER2 phage vaccination. The dosage of palbociclib and treatment schedule were chosen according to recently published data [31].

Blood was collected from the retro-orbital plexus under anesthesia, before and 2 weeks after the first and the second vaccination cycles, and left to clot at room temperature for 20 min. Serum was separated from blood by subsequent centrifugation at 3000 rpm at 4 °C for 10 min and analyzed by FACS. Animals were monitored weekly for tumor onset, growth, and multiplicity by palpation until 26 weeks of age. Progressively growing masses > 1 mm mean diameters were regarded as tumors. Mice were sacrificed when one of the tumors exceeded 10 mm in diameter.

Body weight and food consumption did not significantly differ between treated and control mice, suggesting the absence of a significant systemic toxicity of the administered therapies. At the end of the experiment, mice were euthanized and tumors, heart, kidney and liver samples were collected for subsequent analyses.

### 2.3. Cell Lines

SK-BR-3 cells were cultured in Dulbecco’s Modified Essential Medium (DMEM, CORNING, Mediatech, New York, NY, USA) supplemented with 10% fetal bovine serum (FBS, Gibco, Life Technologies, Carlsbad, CA, USA) and 1% penicillin–streptomycin (P/S) (Gibco, Life Technologies). SK-BR-3 cells were kindly provided by the laboratory of Dr. B. Belletti (Division of Molecular Oncology, CRO of Aviano, IRCCS, National Cancer Institute, Aviano, Italy) and tested for mycoplasma contamination with negative results. CAM3 cells, Δ16HER2-expressing epithelial tumor cell lines established from a mammary carcinoma spontaneously arisen in a Δ16HER2 female [32], were cultured in DMEM supplemented with 20% FBS and 1% P/S. Cells were cultured at 37 °C under a humidified atmosphere with 5% CO_2_.

### 2.4. Analysis of Antibody Response

Sera collected from immunized mice were analyzed by flow cytometry (BD FACSCalibur), using SK-BR-3 cells, to verify the presence of HER2-specific antibodies. Briefly, SK-BR-3 cells were detached and dispensed at a density of 0.5 × 10^6^ cells per tube. After a 5 min centrifugation at 800 rpm at 4 °C, the obtained cell pellet was resuspended and washed twice in staining buffer (2% FBS-containing 1× PBS, pH 7.4, and 0.1% NaN_3_). Cells were incubated with sera of vaccinated mice (1:40 dilution in staining buffer) for 1 h at 4 °C. After incubation, cells were washed three times and incubated on ice in the dark with goat anti-mouse IgG (H+L) secondary antibody-FITC (Thermo Fisher Scientific, Waltham, MA, USA, 1:200 dilution in staining buffer). Samples were washed twice, resuspended in 600 μL of staining buffer and analyzed using BD FACSCalibur. Cell Quest Pro (version 6.0.2) and FlowJo version 8.7 were used as acquisition and analysis software, respectively. Analysis of anti-HER2 antibody titer and identification of different IgG isotypes (IgA, IgM, IgG1, IgG2a, IgG2b, and IgG3) in immune sera (diluted 1:200) was also performed by ELISA. Briefly, sera were incubated on plates coated with human HER2 (Sino Biological, Beijing, China, 100 ng/well) protein, and binding was detected with HRP-conjugated rat anti-mouse-IgG (Merck, Darmstadt, Germany) or with biotinylated rat anti-mouse IgG1, IgG2a, IgG2b or IgG3 (BD Biosciences, San Jose, CA, USA), followed by HRP-conjugated streptavidin. Signals were developed with TMB (Tebu Bio, Paris, France) and analyzed using a 680XR microplate reader (Bio-Rad Hercules, CA, USA). For antibody-dependent cellular cytotoxicity (ADCC), 1 × 10^4^ CAM3 target cells stained with 2 µM CFSE (Molecular Probes, Eugene, Oregon, USA) were cultured overnight with splenocytes from untreated wild-type FVB female mice, used as effector cells, at different effector:target (E:T) ratios (200:1, 100:1, and 50:1 E:T ratio) in the presence of a 1:50 dilution of sera from vaccinated mice. Cells were then collected and stained with 1 μg/mL 7-Amino-ActinomycinD (7-AAD, BD Biosciences), acquired by FACS on a BD FACSVerse and analyzed using Flowjo version 10. ADCC was calculated as described in [33]. In brief, percent killing was obtained by back-gating on the CFSE-positive targets and measuring the % of 7-AAD-positive dead cells. For each serum sample, background killing induced by serum alone (spontaneously dead targets) was measured and subtracted from the killing (dead targets in sample) observed in the presence of the corresponding serum and effector cells. Dead target maximum was obtained after treatment of target cells with Triton X-100 solution. % ADCC for each serum sample was calculated with the formula [(dead targets in sample (%) − spontaneously dead targets (%))/(dead target maximum-spontaneously dead targets (%))] × 100. The control sera used in this assay were obtained from mice treated with empty phage and are therefore referred to as empty phage control sera throughout the manuscript.

### 2.5. Measurements of Cytokines in Sera

The pro- and anti-inflammatory cytokines IL-1α, IL-1β, IL-6, IL-10, IFNγ, and TNF-α were estimated in mouse sera by using multiplex immunoassay (Q-Plex Mouse Cytokine Panel (6-Plex), Quansys Biosciences, Technogenetics Srl., Milan, Italy), Q-View Imager LS, Q-View software Version 3.11, and following the manufacturer’s instructions.

### 2.6. Western Blot Analysis

Tumors were homogenized in RIPA buffer (0.1% SDS, 1% NP40, 0.5% CHAPS) supplemented with protease inhibitors (Sigma-Aldrich, St. Louis, MO, USA). For Western blot analysis, an equal amount of protein lysates was separated onto Criterion™ TGX™ precast gels (4–20% gradient precast SDS-PAGE; Bio-Rad, Hercules, CA, USA) and transferred to a polyvinylidene difluoride (PVDF) membrane (Millipore, Burlington, MA, USA) using Criterion™ Blotter (Bio-Rad, Hercules, CA, USA). Membranes were blocked with EveryBlot Blocking Buffer (Bio-Rad, Hercules, CA, USA) and then incubated overnight with primary antibodies at 4 °C. Primary antibodies to vinculin (sc-73614) and HER2 (sc-284, lot #I0507) were from Santa Cruz Biotechnology (Dallas, Texas, USA). Primary antibody to phospho-Rb (Ser780) (C84F6, lot 3) was from Cell Signaling (Danvers, MA, USA). Secondary antibodies conjugated with peroxidase were from Sigma-Aldrich (Sigma-Aldrich/Merck, Darmstadt, Germany). Secondary antibody binding was performed at room temperature for 1 h. After TBS-T washing, membranes were incubated with PierceTM ECL Western blotting Substrate (Thermo Scientific, Boston, MA, USA), and the immunoreactive proteins were detected with ChemiDoc™ XRS-System (Bio-Rad, Hercules, CA, USA). Densitometry analysis was performed through ImageJ software (Version: 2.1.0/1.53C).

### 2.7. Purification of IgG

IgG were purified from sera of ECTM-phage vaccinated or empty phage treated (control) mice using the Melon Gel IgG Purification Kit (Thermo Fisher Scientific, Waltham, MA, USA) and quantified using a NanoDrop spectrophotometer using IgG settings (Thermo Fisher Scientific, Waltham, MA, USA).

### 2.8. Cell Viability Assay

SK-BR-3 cells (1 × 10^4^ cells/well) were seeded in 96-well plates. The day after, fresh medium (DMEM plus 2% FBS), containing appropriate IgG, palbociclib or trastuzumab (HY-P9907, MedChemExpress, Princeton, NJ, USA) concentrations, was added. Cell viability was determined after 72 h using an MTT (3-(4,5-dimethylthiazol-2-yl)-2,5-diphenyl-2H-tetrazolium bromide, Sigma Aldrich/Merck, Darmstadt, Germany) assay, which is based on the conversion of MTT to formazan by mitochondrial enzymes. The formazan deposits were dissolved in dimethyl sulfoxide (DMSO), and the absorbance of each well was measured at 540 nm in Multiskan Ascent 96/384 Plate Reader (Thermo Fisher, Helsinki, Finland). Each drug concentration was evaluated with six replicates and the experiments were repeated three times.

### 2.9. Histological Analysis

Heart, kidney and liver samples were fixed in 4% neutral buffered paraformaldehyde solution and embedded in paraffin. Slides were cut and stained with Hematoxylin (Bio-Optica, Milan, Italy) and Eosin (Bio-Optica, Milan, Italy)) for histological examination. Sections of the heart were processed for Picro-Sirius Red (Direct Red 80, Sigma Aldrich), used for histological visualization of collagen I and III fibers to evaluate the fibrotic process as previously described [34]. The sections were observed, and images were captured with the microscope by a DS-Ri2 NIKON camera (Niko, Florence, Italy), and the area of fibrosis around the cardiomyocyte was measured using a NIS Elements Nikon image analyzer software (4.30.02 64).

### 2.10. Immunohistochemical and Immunofluorescence Analyses

Tumor samples were fixed in 10% neutral buffered formalin and embedded into paraffin; slides were cut and stained with Hematoxylin (Bio-Optica, Milan, Italy) and Eosin (Bio-Optica, Milan, Italy) for histological examination. For immunohistochemistry or immunofluorescence, slides were deparaffinized, serially rehydrated, and, after the appropriate antigen retrieval procedure, stained with the following primary antibodies: rabbit anti-mouse CD3 antibody (ab16669, Abcam, Cambridge, UK), rabbit anti-mouse CD8 antibody (98941, Cell signaling, Danvers, MA, USA), rat anti-mouse FOXP3 antibody (14-5773-82, e-Bioscience, San Diego, CA, USA), and rabbit anti-human HER2 antibody (A0485, Dako, Glostrup, Denmark). Immunostainings were developed with streptavidin peroxidase methods and the DAB Chromogen system (Dako, Glostrup, Denmark). After chromogen incubation, slides were counterstained in Hematoxylin (Bio-Optica, Milan, Italy). For the evaluation of CD8-positive cells, whole-tumor sections were scanned using a Nanozoomer scanner (Hamamatsu Photonics, Hamamatsu, Shizuoka Prefecture, Japan) and analyzed using Qu-Path software (v0.3.2.). For immunofluorescence, immunostainings were developed using anti-rabbit Alexa Fluor 488 (A11008, ThermoFisher, Waltham, MA, USA) and anti-rat Alexa Fluor 546 (A11081, ThermoFisher, Waltham, MA, USA) as secondary antibodies. Nuclei were stained with Dapi (Sigma-Aldrich/Merck, Darmstadt, Germany). HER2 protein expression was evaluated by measuring the Mean Fluorescence Intensity (MFI) with NIS-Elements software on digital images of 4–5 tumors per group (analyzing 5 microscopic fields at 200× magnification per sample). Images were acquired with a Zeiss LSM800 (Carl Zeiss Microscopy GmbH, Oberkochen, Germany) or a Nikon AXR Nspark (Nikon, Florence, Italy) confocal microscope.

### 2.11. Microbial Community Analysis of the Mouse Gut Microbiome

Mouse feces (250–300 mg per each mouse) were dissolved in 0.5 mL of the SLX buffer supplied by the E.Z.N.A. Kit (Omega Bio-tek Inc., Norcross, GA, USA). After the addition of glass beads, samples were homogenized by vortexing for 10 min. Chromosomal DNA extraction was then completed according to the instructions given by the kit manufacturer. DNA concentration was estimated by NanoDrop (ThermoFisher Scientific, Waltham, MA, USA). The V3–V4 hypervariable regions of 16S rDNA were amplified by PCR using universal primers 341F 5′-TCGTCGGCAGCGTCAGATGTGTATAAGAGACAGCCTACGGGNGGCWGCAG-3′ and 805R 5′-GTCTCGTGGGCTCGGAGATGTGTATAAGAGACAGGACTACHVGGGTATCTAATCC-3′ and amplicon libraries were sequenced on an Illumina MiSeq platform (Illumina Inc., San Diego, CA, USA) using a 2 × 250 bp paired-end sequencing and following the manufacturer’s standard protocols [35]. Briefly, raw sequencing reads were subjected to quality control to remove low-quality bases, adapter contamination, and short reads using Trimmomatic. High-quality paired-end reads were subsequently merged into full-length amplicon sequences using FLASH [36], based on the overlap between forward and reverse reads. The resulting merged sequences were further processed with VSEARCH to identify and remove potential chimeric sequences. After chimera filtering, sequences were clustered into Operational Taxonomic Units (OTUs) at a 97% sequence similarity threshold [37]. The NCBI 16S RefSeq [38] database was employed for taxonomic classification.

### 2.12. Statistical Analyses

Statistical analysis was carried out with GraphPad Prism 10 Software (San Diego, CA, USA), using the most appropriate test, as specified in each figure. *p* < 0.05 was used as the critical level of significance.

## 3. Results

### 3.1. Therapeutic Anti-HER2 Phage-Based Vaccination Delays Tumor Onset but Does Not Maintain Long-Term Control

**Figure 1:**
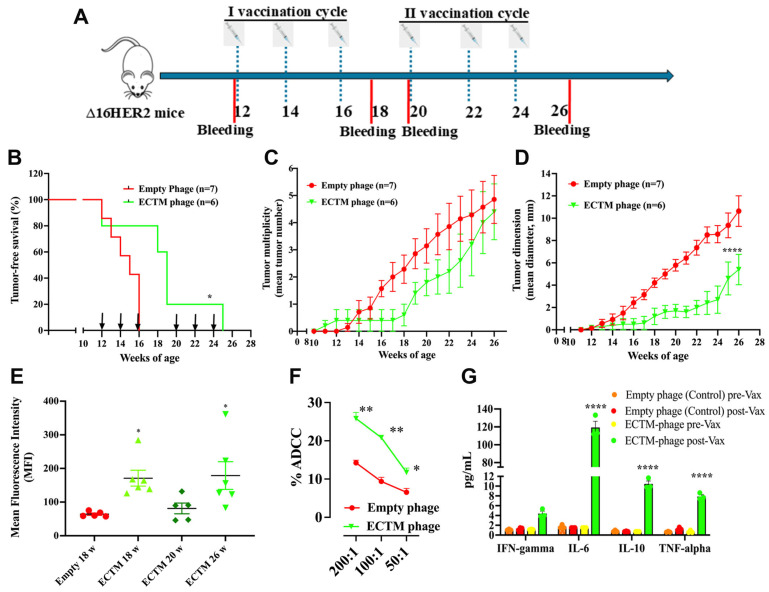
Therapeutic phage-based vaccination (protocol 3 × 2) against HER2+/HR+ breast cancer in Δ16HER2 mice. (**A**). Experimental workflow: ECTM-phages (1 × 10^11^ CFU/mouse) were injected intraperitoneally (i.p.) at 12, 14, 16 weeks of age (first vaccination cycle), and at 20, 22, and 24 weeks of age (second vaccination cycle). Empty phages were used as control. Bleeding occurred at the indicated times for antibody analysis. The number (n) of mice is indicated at the top of panels (**B**–**D**). (**B**). Kaplan–Meier curves; Log Rank test (* *p* < 0.05 ECTM versus (vs.) empty phages (control)). (**C**). Tumor multiplicity curves. (**D**). Tumor growth curves; **** *p* < 0.0001 empty phages (control) vs. ECTM-phages. (**E**). Antibody detection. Sera were collected from ECTM-phage vaccinated or empty phage (control) treated mice at the indicated times and analyzed by FACS using SK-BR-3 cells; data represent Mean fluorescence intensity (MFI) ± SE; One-way ANOVA with Dunnett’s multiple comparison test (* *p* < 0.05 empty phages (control) vs.ECTM-phages (two weeks after the first vaccination cycle); * *p* < 0.05 empty phages (control) vs. ECTM-phages (two weeks after the second vaccination cycle)) (n = 5–6). (**F**). ADCC assay was performed using CAM3 target cells incubated with a 1:50 dilution of sera from ECTM-phage vaccinated or empty phage (control) treated mice (harvested at 26 weeks of age; n = 6) and splenocytes as effector cells at different effector/target cell ratios (200:1, 100:1 and 50:1). Serum-independent target killing was 2.8% at 200:1, 1.2% at 100:1, and 0% at 50:1. Results are shown as the mean ± SEM of the percentage of ADCC induced by different sera; Student’s *t*-test (* *p* < 0.05; ** *p* < 0.01). (**G**). Quantification of cytokines in mouse sera by using a multiplex immunoassay. Sera were harvested from empty phage (control) and ECTM-phage vaccinated mice, before immunization and two weeks after the second vaccination cycle. Data are shown as mean ± SEM (n = 3). Unpaired *t*-test, empty phages (control) vs. ECTM-phage post-Vax (IL-6: *p* = 0.000069; IL-10 *p* = 0.000091; TNF-a *p* = 0.000095; IFN-g *p* = 0.003474). **** *p* < 0.0001.

We first evaluated the therapeutic efficacy of an optimized anti-HER2 phage-based vaccination schedule in Δ16HER2 transgenic female mice, which spontaneously develop multiple HR^+^/HER2^+^ mammary adenocarcinomas with a 100% penetrance starting at 12 weeks of age [15,39]. ECTM-phages displaying the EC and TM domains of human HER2 on M13 virions were administered intraperitoneally according to a 3 × 2 protocol, consisting of three doses at 12, 14 and 16 weeks of age followed by three additional doses at 20, 22 and 24 weeks (1 × 10^11^ CFU/mouse per injection), while empty phages were used as control (Figure 1A). This protocol significantly delayed tumor onset and reduced tumor growth rate compared with control mice. At 16 weeks of age, 80% of vaccinated mice were tumor-free, whereas all empty phage-treated (control) mice bore at least one palpable tumor (Figure 1B). Tumor multiplicity and mean tumor diameter were also lower in ECTM-vaccinated animals at 16–18 weeks, indicating a marked therapeutic effect (Figure 1C,D). However, this protection was not durable: by 20 weeks of age only 20% of vaccinated mice remained tumor-free, and by 25 weeks all immunized animals had developed multiple tumors, with tumor multiplicity no longer efficiently controlled (Figure 1B,C). Analysis of the humoral response showed that the first vaccination cycle induced a robust increase in anti-HER2 antibodies, as measured by flow cytometry on SK-BR-3 cells, with titers peaking two weeks after the third dose and declining thereafter (Figure 1E). The second vaccination cycle boosted anti-HER2 antibody levels again and the sera from vaccinated mice were able to mediate ADCC against Δ16HER2-derived CAM3 cells in vitro (Figure 1F), which was correlated with tumor growth inhibition in individual mice (Figure S1). Multiplex cytokine analysis of sera collected two weeks after the second vaccination cycle revealed increased levels of IFN-γ and TNF-α, together with elevated IL-10 and IL-6, in ECTM-vaccinated mice compared with controls (Figure 1G).

### 3.2. Combination of ECTM-Phage Vaccination with Palbociclib Enhances Tumor Control and Modulates HER2 Signaling

**Figure 2:**
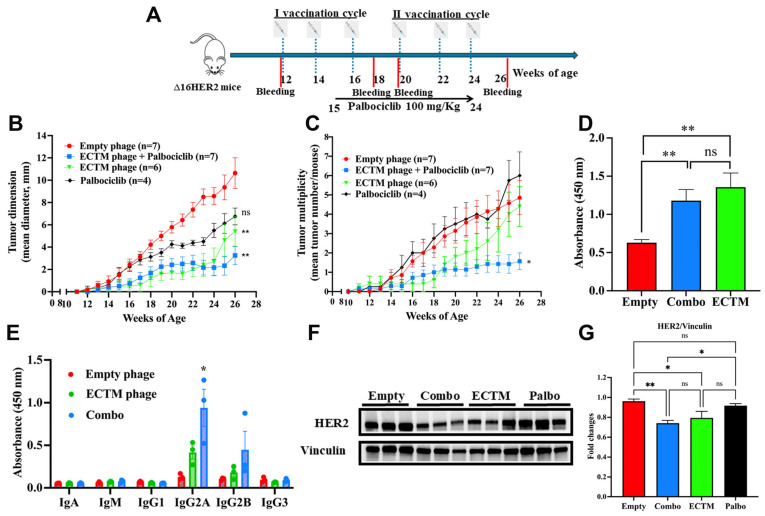
Therapeutic phage-based vaccination in combination with palbociclib against HER2+/HR+ breast cancer in Δ16HER2 mice. (**A**). Experimental workflow: palbociclib (100 mg/Kg) was administered daily by oral gavage from 15 to 24 weeks of age; ECTM-phages (1 × 10^11^ CFU/mouse) were injected intraperitoneally (i.p.) at 12, 14, 16 weeks of age (first vaccination cycle), and at 20, 22, and 24 weeks of age (second vaccination cycle), alone or in combination with palbociclib (combo); empty phages were used as control. Bleeding occurred at the indicated times for antibody analysis. The number (n) of mice is indicated at the top of panels (**B**,**C**). (**B**). Tumor growth curves; One-way ANOVA with Tukey’s multiple comparison test (** *p* < 0.01 empty phage (control) vs. ECTM phages; ** *p* < 0.01 empty phage (control) vs. combo). (**C**). Tumor multiplicity curves; One-way ANOVA with Tukey’s multiple comparison test (* *p* < 0.05 combo *vs.* empty phage (control); ). (**D**). Anti-HER2 antibody detection in mice vaccinated with ECTM-phages, alone or in combination with palbociclib (combo), or treated with empty phage (control). Sera of mice collected at 26 weeks of age were diluted 1:200 and analyzed by ELISA; data are expressed as mean ± SEM (n = 6); Student’s *t*-test (** *p*< 0.01 empty phage (control) vs. ECTM phages; ** *p* < 0.01 empty phage (control) vs. combo; ns: not significant). (**E**). Analysis of antibody isotypes induced by vaccination with ECTM-phages alone or in combination with palbociclib (combo) (n = 3 mice per group) by ELISA. Sera were collected from mice after the second vaccination cycle (at 26 weeks of age) and diluted 1:200. Data are expressed as mean ± SEM. One-way ANOVA test followed by Tukey’s multiple comparison test (* *p* < 0.05 empty phage (control) vs. combo). (**F**). Representative Western blot analysis of HER2 and vinculin (loading control) in three different tumors explanted from 30-week-old Δ16HER2 mice receiving the indicated treatments (n = 3 mice/group). Equal amounts of protein (20 μg) were loaded. (**G**). Densitometric quantification of HER2 expression normalized with vinculin from two independent experiments is shown. Data are expressed as mean ± SEM. One-way ANOVA test followed by Tukey’s multiple comparison test (* *p* ≤ 0.05; ** *p* ≤ 0.01; ns: not significant).

We next investigated whether combining ECTM-phage vaccination with the CDK4/6 inhibitor palbociclib could improve therapeutic efficacy in Δ16HER2 mice. Palbociclib was administered by oral gavage at 100 mg/kg/day, 5 days per week, from 15 to 24 weeks of age, either alone or in combination with the ECTM vaccination following the 3 × 2 schedule; empty phages were used as control (Figure 2A). The start of palbociclib treatment was delayed relative to the first vaccine dose to avoid interference with T-cell expansion [40]. Treatment with palbociclib alone did not significantly reduce tumor growth rate or tumor multiplicity compared with control mice (Figure 2B,C). In contrast, the combination of palbociclib with ECTM-phage vaccination resulted in a significantly better and more durable control of both tumor growth and multiplicity than either monotherapy. Tumor diameters in the combination group remained significantly lower than those in empty phage (control) and palbociclib-treated mice throughout the observation period, and tumor multiplicity was significantly reduced in the combination group compared with both empty phage (control) and palbociclib alone groups (Figure 2B,C). Measurement of anti-HER2 antibodies by ELISA in sera collected at 26 weeks of age showed that total anti-HER2 IgG titers were comparably increased in mice vaccinated with ECTM-phages alone and in those receiving the combination therapy, relative to empty-phage controls (Figure 2D). By contrast, analysis of IgG subclasses revealed that the combination regimen induced a higher proportion of IgG2a isotype antibodies compared with vaccination alone, whereas other isotypes were not markedly altered (Figure 2E). Western blot analysis showed that total HER2 levels were reduced in tumors explanted from mice receiving ECTM-phage vaccine alone or in combination with palbociclib with respect to empty phage (control) mice, suggesting that anti-HER2 antibodies induced by vaccination could promote HER2 internalization and degradation (Figure 2F,G).

**Figure 3:**
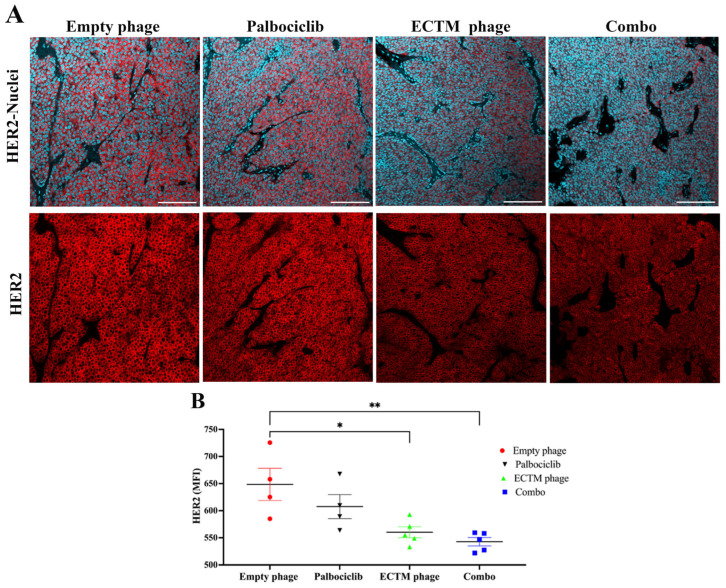
HER2 analysis on tumor sections by confocal microscopy. (**A**). Representative images of tumor sections: HER2 (red) was stained in tumors explanted from Δ16HER2 mice receiving empty phages (control), or palbociclib, or ECTM-phages alone, or ECTM-phages in combination with palbociclib (combo). Nuclei are stained with DAPI (blue). Scale bar, 100 μm. (**B**). Quantification of HER2 in sections of tumors explanted from Δ16HER2 mice receiving the indicated treatments. One-way ANOVA test followed by Tukey’s multiple comparison test. ** *p* < 0.01 combo vs. empty phage (control); * *p* < 0.05 ECTM-phage vs. empty phage (control). Data are expressed as mean ± SEM.

These results were confirmed by surface HER2 staining by immunofluorescence on tumor sections and quantitative confocal microscopy analysis. Indeed, a statistically significant reduction in HER2 protein levels was detected following treatment with ECTM phage (*p* = 0.0174) in comparison with the empty phage control and an even more pronounced HER2 decrease was observed when ECTM-phage vaccine was combined with palbociclib (combo group (*p* = 0.0064)) (Figure 3). In parallel, decreased levels of phospho-Rb were detected in tumors explanted from mice treated with palbociclib, alone or in combination with ECTM-phage vaccine, confirming the CDK4/6 targeted inhibition (Figure S2). Moreover, the functional activity of anti-HER2 IgG antibodies, purified from sera of mice vaccinated with ECTM-phages, was tested in vitro using SK-BR-3 cells. Anti-HER2 IgG antibodies were able to reduce SK-BR-3 cell viability when administered singularly and importantly enhanced the efficacy of palbociclib, indicating that their combination was significantly more effective than monotherapies (Figure S3). Thus, these results suggest that the efficacy of the proposed therapeutic strategy is mediated by a direct and antigen-specific effect on HER2-positive breast cancer cells.

To evaluate the toxicity of palbociclib and ECTM-phage vaccination, administered either as monotherapy or in combination, we carried out histopathological analyses of explanted kidneys, liver, and heart, common target organs of anticancer drugs. Histopathological analysis of liver (Figure S4A) and kidney (Figure S4B) tissues did not reveal any parenchymal alterations across the different experimental groups. Analysis of heart sections from ECTM-phage vaccinated mice did not show any signs of significant cardiotoxicity. However, heart sections from Δ16HER2 mice treated palbociclib, either alone or in combination with ECTM-phage vaccine, exhibited an increased fibrotic area characterized by collagen fiber deposition compared with control or ECTM-phage vaccinated mice (Figure S5A,B).

### 3.3. Combination Therapy Reshapes Intratumoral CD8^+^ T-Cell and Treg Compartments

**Figure 4:**
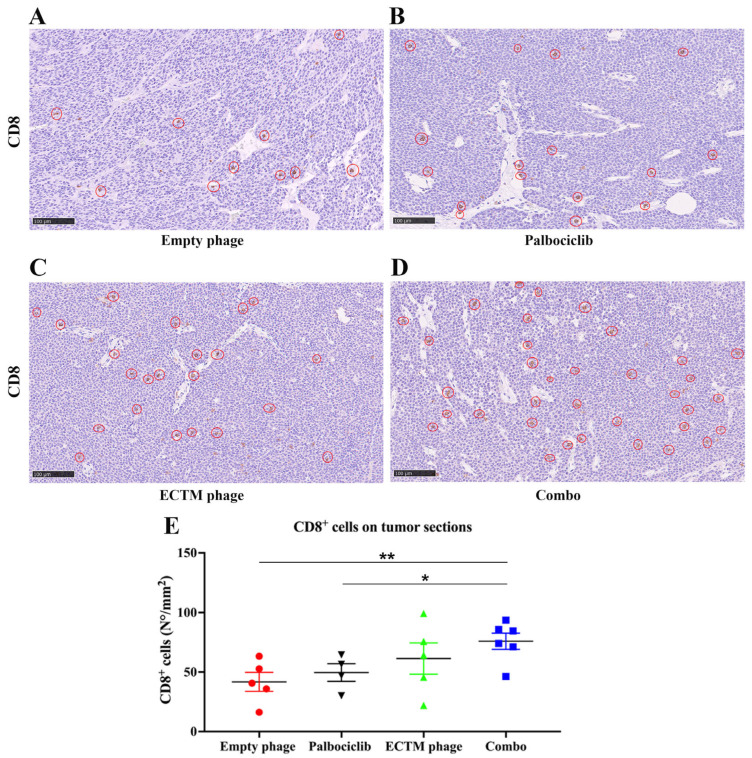
CD8^+^ T cells evaluation on tumor sections by immunohistochemistry. Representative images of tumor sections: CD8^+^ T lymphocytes (red circle) were stained in tumors explanted from Δ16HER2 mice receiving empty phages (control) (**A**), or palbociclib (**B**) or ECTM-phages alone (**C**) or ECTM-phages in combination with palbociclib (combo) (**D**). Scale bar, 100 μm; original magnification, ×40. (**E**). Quantification of CD8^+^ T cells in sections of tumors explanted from Δ16HER2 mice receiving the indicated treatments. ** *p* < 0.01 combo vs. empty phage (control); * *p* < 0.05 combo vs. palbociclib). Data are expressed as mean ± SEM; n = 4–6.

To characterize the immune correlates of treatment efficacy, we analyzed tumor-infiltrating lymphocytes by immunohistochemistry and immunofluorescence. Immunostaining for CD8^+^ cells showed that tumors from mice treated with ECTM-phages or palbociclib alone displayed a modest increase in CD8^+^ T-cell infiltration compared with controls, whereas tumors from mice receiving the combination therapy exhibited a marked enrichment of CD8^+^ T cells within the tumor microenvironment (Figure 4A–D). Quantitative analysis confirmed that CD8^+^ T-cell density was significantly higher in the combination group than in control and palbociclib-treated mice (Figure 4E).

**Figure 5:**
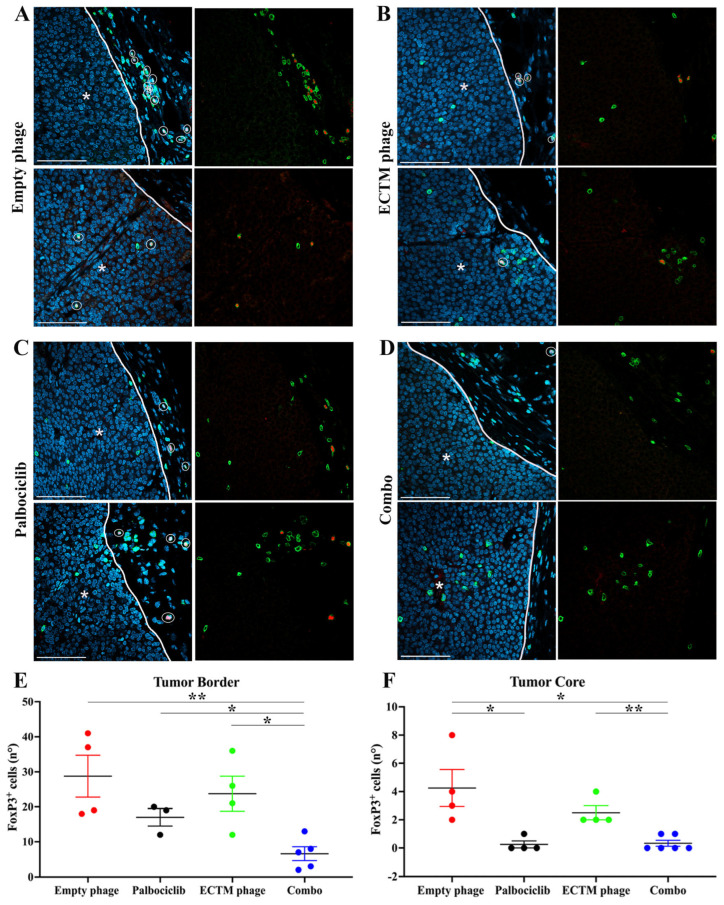
Evaluation of Treg abundance in tumors by immunofluorescence. Representative images of immunofluorescence analysis of T lymphocytes and Tregs, stained for membranous CD3 (green) and nuclear Foxp3 (red), in tumors explanted from Δ16HER2 mice receiving empty phage (control) (**A**), ECTM-phage vaccine (**B**), palbociclib (**C**), or the combination therapy (combo) (**D**) (n = 4–6 tumors/group). Both peritumoral (upper panels) and intratumoral (lower panels) areas were analyzed and quantified (**E**,**F**). In the (**A**–**D**) left panels, the tumor border is indicated by the white line; the tumor core is indicated by the white asterisk, and Treg cells are circled; nuclei are stained with DAPI (blue). Scale bar 50 μm. (* *p* ≤ 0.05; ** *p* ≤ 0.01).

Treg cells were evaluated by confocal microscopy as CD3^+^FoxP3^+^ cells in peritumoral and intratumoral areas. In control tumors, Treg cells were mainly localized in the stromal area at the tumor border, with some infiltration into the tumor core (Figure 5A). ECTM-phage vaccination did not substantially reduce Treg abundance at the tumor margins but decreased the number of Treg cells within the tumor parenchyma (Figure 5B). Palbociclib treatment slightly reduced Treg cells at the tumor border and more markedly within the tumor core (Figure 5C). Notably, the combination of ECTM-phages and palbociclib led to a strong depletion of Treg cells, with only a few CD3^+^FoxP3^+^ cells detectable at the tumor border and virtually none within the tumor core (Figure 5D).

### 3.4. Anti-HER2 ECTM-Phage Vaccination Selectively Modulates the Gut Microbiome

**Figure 6:**
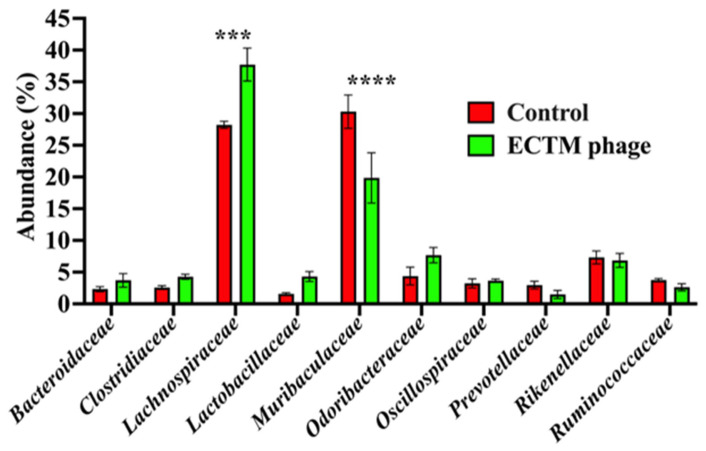
Impact of ECTM-phage vaccination on gut microbiome. The abundance of bacterial families in the gut microbiome of control or ECTM-phage-vaccinated mice is shown. DNA was extracted from the fecal matter of individual mice (n = 4/group) left untreated (control) or immunized with ECTM-phage, and species identification was carried out by 16S rRNA gene next-generation sequencing (NGS). Data are expressed as mean ± SEM. Two-way ANOVA followed by Šidák’s multiple comparison test (*** *p* < 0.001; **** *p* < 0.0001 control vs. ECTM-phage-immunized group).

Finally, we assessed the impact of ECTM-phage vaccination on the gut microbiome. 16S rRNA gene next-generation sequencing of fecal samples from control and vaccinated mice (n = 4 per group) showed that ECTM-phage treatment did not markedly alter overall bacterial family richness or the relative abundance of most families (Figure 6). However, vaccinated mice displayed a significant increase in the relative abundance of *Lachnospiraceae* and a concomitant decrease in *Muribaculaceae* compared with controls (Figure 6).

## 4. Discussion

Here, we show that CDK4/6 inhibition can enhance the therapeutic durability of anti-HER2 vaccination and reshape the tumor immune microenvironment, overcoming the tumor-induced immunosuppression. Indeed, the combination of a phage-based anti-HER2 vaccine with the CDK4/6 inhibitor palbociclib resulted in a long-lasting control of mammary tumor growth in Δ16HER2 transgenic mice, associated with enhanced intratumoral CD8^+^ T-cell infiltration and a profound depletion of regulatory T cells (Treg), while a robust anti-HER2 humoral response was preserved.

### 4.1. Phage-Based Anti-HER2 Vaccination as a Pharmaceutically Relevant Platform

From a pharmaceutics perspective, filamentous M13 bacteriophages represent a versatile vaccine platform that combines high antigen density, intrinsic immunogenicity, and relative ease of large-scale production using *Escherichia coli* hosts. In our model, ECTM-phages displaying the extracellular and transmembrane domains of human HER2 [17,18] elicited robust HER2-specific antibody responses and significantly delayed tumor onset in Δ16HER2 mice, which are immunotolerant to human HER2 and spontaneously develop HR^+^/HER2^+^ mammary carcinomas [15,16,17,39]. These findings confirm and extend previous evidence that phage-based vaccines can break tolerance against self-tumor antigens and provide therapeutic benefit in established disease. However, despite optimization of the vaccination schedule and dose (3 × 2 protocol with increased phage load), ECTM-phage vaccination alone was ultimately unable to maintain long-term tumor control, as all immunized mice eventually developed multiple tumors. The kinetic analysis of the humoral response showed that anti-HER2 antibody titers peaked shortly after the first vaccination cycle and then declined, with a partial boost following the second cycle, paralleled by changes in pro- and anti-inflammatory cytokines. This transient pattern suggests that, in the context of an advanced, immunosuppressive tumor microenvironment, active vaccination alone is insufficient to sustain durable immune pressure on tumor cells. These observations underscore both the potential and the limitations of phage-based cancer vaccines as pharmaceutically relevant immunotherapeutics: while they offer an attractive, modular platform for antigen delivery, their full therapeutic impact in established tumors is likely to require rational combination with agents that recondition the host immune milieu.

### 4.2. CDK4/6 Inhibition Cooperates with Vaccination to Reprogram the Tumor Immune Microenvironment

CDK4/6 inhibitors were initially developed as cell cycle-targeted agents, but accumulating evidence indicates that they exert broad immunomodulatory effects, including enhancement of antigen presentation, promotion of memory-like CD8^+^ T cells, and selective inhibition of Treg cell proliferation [23,24,25]. In our study, palbociclib administered as monotherapy at a clinically relevant schedule did not significantly reduce tumor growth or multiplicity compared with controls in Δ16HER2 mice, representing an aggressive breast cancer preclinical model. Nonetheless, palbociclib alone modestly increased intratumoral CD8^+^ T-cell infiltration and reduced Treg abundance within the tumor core, in line with its reported capacity to modulate T-cell subsets [23]. When palbociclib was combined with ECTM-phage vaccination, these immune effects were markedly amplified. Tumors from mice receiving the combination therapy displayed an enrichment of CD8^+^ T cells and a depletion of CD3^+^FoxP3^+^ Treg cells at both the tumor border and within the tumor parenchyma. This shift in the effector/Treg balance was accompanied by a qualitative change in the humoral response, with a higher proportion of anti-HER2 IgG2a antibodies in the combination group compared with vaccination alone, an isotype associated with enhanced Fc-mediated effector functions in therapeutic settings [41]. Overall, these data support a model in which palbociclib creates a more permissive immune landscape by limiting Treg-mediated suppression and promoting CD8^+^ T-cell infiltration in which phage-induced anti-HER2 effector responses can be sustained and efficiently translated into superior tumor control. This cooperative interaction between cell cycle inhibition and active vaccination highlights the potential of CDK4/6 inhibitors as immunomodulatory partners for cancer vaccines, beyond their direct antiproliferative effects.

Analysis of organ toxicity revealed that both the CDK4/6 inhibitor palbociclib and ECTM phage vaccination were well tolerated, with no evidence of liver or kidney damage, supporting the acceptable safety profile of both therapeutic approaches. Of note, phage-based anti-HER2 vaccination was not associated with cardiotoxicity. In contrast, treatment with palbociclib, either alone or in combination with ECTM phage vaccination, was associated with increased cardiomyopathy characterized by collagen fiber deposition between cardiomyocytes. This finding may be related to the cardiovascular toxicity of CDK4/6 inhibitors previously reported in women with breast cancer undergoing pharmacological treatment [42]. Indeed, although CDK4/6 inhibitors are generally considered safe, several studies have reported cardiotoxic effects associated with palbociclib, ribociclib and abemaciclib, alone or combined with endocrine therapy [42,43]. Reported adverse events include arrhythmias, hypertension, atrial fibrillation, myocardial infarction, vascular inflammation, heart failure, atrioventricular block, QT interval prolongation and increased Fridericia-corrected QT values. However, other clinical studies found no significant cardiac toxicity, with preserved cardiac function and stable left ventricular ejection fraction [43]. The mechanisms underlying CDK4/6 inhibitor-induced cardiotoxicity remain unclear. Current evidence suggests that multiple interconnected pathways, like those described for other kinase inhibitors, may contribute to myocardial injury. Indeed, preclinical studies of kinase inhibitors have consistently demonstrated reduced left ventricular function, myocardial fibrosis, oxidative stress, mitochondrial dysfunction and cardiomyocyte apoptosis [44]. On the other hand, our findings indicate that phage vaccination neither induces nor exacerbates cardiac alterations caused by palbociclib.

### 4.3. Exploratory Evidence for Microbiome Modulation by Phage-Based Vaccination

An additional, exploratory aspect of our work concerns the impact of anti-HER2 phage vaccination on the gut microbiome. This part of the study did not include palbociclib as the main objective was to exclude harms to the microbiome caused by the phage-based vaccine. ECTM-phage immunization did not markedly alter overall microbiome diversity or the relative abundance of most bacterial families, indicating that systemic administration of bacteriophages at the tested dose and schedule does not grossly disrupt gut microbial communities. However, we observed a selective increase in the relative abundance of *Lachnospiracea* and a decrease in *Muribaculaceae* in vaccinated mice compared with controls. Notably, *Lachnospiraceae* have been reported to be enriched in responders to Trastuzumab in HER2-positive breast and gastrointestinal cancers, suggesting a potential link between this family and improved outcomes of HER2-targeted therapies [28,29]. Conversely, *Muribaculaceae* have been implicated in pro-inflammatory states [45], although their role in tumor immunity remains incompletely understood. Given the small sample size and the observational nature of the analysis, these findings should be interpreted as hypothesis-generating and do not allow causal inferences about the contribution of specific taxa to the efficacy of vaccination. Nevertheless, they raise the intriguing possibility that phage-based vaccines could exert dual immunomodulatory actions by directly priming anti-HER2 immune responses and indirectly shaping the gut microbiota in a manner that may favor antitumor immunity. Future studies integrating metagenomic and metabolomic profiling with longitudinal immune monitoring will be essential to clarify the interplay between bacteriophage-based vaccination, alone or in combination with CDK4/6 inhibitors, and the gut microbiome.

### 4.4. Limitations of the Study

This study has several limitations that should be acknowledged. First, all experiments were conducted in a single transgenic mouse model of HR^+^/HER2^+^ breast cancer, which, although highly relevant and well characterized, cannot fully recapitulate the molecular and immunological heterogeneity of human HER2-positive tumors. Second, the ADCC assay was performed using total effector cell preparations rather than purified NK cells or FcγR-expressing reporter cells. Therefore, although the increase in IgG2a and the serum-dependent killing observed in vitro are consistent with enhanced Fc-mediated effector potential, additional experiments using purified NK cells, FcγR-reporter assays, or FcγR-blocking approaches will be required to definitively establish the contribution of FcγR-dependent mechanisms to tumor control in vivo. Third, we focused on a single CDK4/6 inhibitor (palbociclib) and one dosing schedule; whether other CDK4/6 inhibitors with distinct pharmacokinetic and pharmacodynamic profiles, or alternative sequencing regimens, might further optimize the balance between tumor cell-cycle arrest and T-cell expansion remains to be determined. Finally, the microbiome analysis was exploratory and limited to 16S rRNA gene sequencing at the family level in a small number of animals, precluding definitive conclusions about the biological significance of the observed compositional changes. Larger cohorts, higher-resolution microbiome profiling, and interventional approaches (e.g., fecal microbiota transfer or antibiotic modulation) will be required to validate the impact of phage-based vaccination on gut microbial communities and their relevance for antitumor immunity.

### 4.5. Translational Implications and Pharmaceutics Perspective

Despite these limitations, our findings have important translational implications. Palbociclib is already approved for HR^+^/HER2^−^ breast cancer and is under clinical evaluation in combination with HER2-targeted therapies in HER2-positive disease, providing a clinically tractable backbone for combinatorial strategies [46]. Our data suggest that integrating a phage-based anti-HER2 vaccine into CDK4/6 inhibitor–containing regimens may offer a means to induce durable, antigen-specific immune memory while simultaneously alleviating tumor-driven immunosuppression. This is a crucial point, considering that up to now clinical trials evaluating breast cancer vaccines have provided limited evidence of clinical benefits mainly due to the immune-suppressive microenvironment [47]. From a pharmaceutics standpoint, the use of filamentous phages as modular, genetically programmable vaccine carriers opens opportunities for scalable manufacturing, rapid antigen redesign, and flexible combination with existing targeted agents. Collectively, these results provide a preclinical rationale for the clinical translation of CDK4/6 inhibitors in combination with phage-based anti-HER2 active immunotherapies and motivate further investigation into the role of the gut microbiome as a potential modulator of treatment outcome. However, considering that palbociclib might be associated with an increased risk of cardiovascular toxicity, a comprehensive risk–benefit assessment is required before initiating this treatment in breast cancer patients. Furthermore, periodic monitoring of cardiac function during the first year after treatment completion is strongly recommended, together with a long-term surveillance plan that includes a healthy lifestyle, a balanced diet, regular monitoring of blood pressure and cholesterol levels, smoking cessation, and stress management [48].

## Data Availability

The data presented in this study are available in this article and Supplementary Material. Further inquiries can be directed to the corresponding authors.

## Supplementary Materials

The following supporting information can be downloaded at: https://www.mdpi.com/article/10.3390/pharmaceutics18070881/s1.
