# Supplementary material for: Targeting CDK4/6 in Combination with Phage-Based Anti-HER2 Vaccination Overcomes Immune Evasion and Enhances the Anticancer Response in Breast Cancer
Source: Pharmaceutics. 2026 Jul 18;18(7):881. doi: 10.3390/pharmaceutics18070881 (PMC13414912; doi:10.3390/pharmaceutics18070881)
Supplement: Supplementary file 1 [file pharmaceutics-18-00881-s001.zip › pharmaceutics-4389435-supplementary.pdf]

SUPPLEMENTARY INFORMATION

Targeting CDK4/6 in combination with phage-based anti-HER2 vaccination overcomes immune evasion and enhances the anticancer response in breast cancer

Junbiao Wang [1, †], Alessia Lamolinara [2, †], Daniele Tomassoni [1], Laura Conti [3], Chiara Cossu [3], Antonino Di Lorenzo [3], Mara Giangrossi [1], Daniela Lufrano [1,8], Varshini Vaithianathan [1], Fiorenza Orlando [4], Fabiola Olivieri [5, 6], Serena Marcozzi [6], Daniela Beghelli [1], Barbara Belletti [7], Augusto Amici [1], Maurizio Falconi [1, *], Federica Cavallo [3], Manuela Iezzi [2, ‡], Cristina Marchini [1, ‡, *]

[1] School of Biosciences and Veterinary Medicine, University of Camerino, via Gentile III da Varano, 62032 Camerino, Italy; junbiao.wang@unicam.it; daniele.tomassoni@unicam.it; mara.giangrossi@unicam.it; daniela.lufrano@unicam.it; daniela.beghelli@unicam.it; varshi.vaithianathan@studenti.unicam.it; augusto.amici@unicam.it; maurizio.falconi@unicam.it; cristina.marchini@unicam.it

[2] Center for Advanced Studies and Technology, Department of Neurosciences, Imaging and Clinical Sciences, G. d'Annunzio University of Chieti-Pescara, 66013 Chieti, Italy; alessia.lamolinara@unich.it; miezzi@unich.it

[3] Department of Molecular Biotechnology and Health Sciences, Molecular Biotechnology Center "Guido Tarone", University of Torino, 10126 Torino, Italy; laura.conti@unito.it; federica.cavallo@unito.it

[4] Experimental Animal Models for Aging Unit, Scientific Technological Area, IRCCS INRCA, 60100 Ancona, Italy; F.ORLANDO@inrca.it

[5] Department of Clinical and Molecular Sciences, UniversitàPolitecnica Delle Marche, 60100 Ancona, Italy; f.olivieri@staff.univpm.it

[6] Biogerontology Center and Geriatric Mouse Clinic, IRCCS INRCA, 60121 Ancona, Italy; S.MARCOZZI@inrca.it

[7] Molecular Oncology Unit, Centro di Riferimento Oncologico di Aviano (CRO Aviano), IRCCS, National Cancer Institute, 33081 Aviano, Italy; bbelletti@cro.it

[8] Department of Biological Sciences, Faculty of Exact Sciences, National University of La Plata, CONICET (Consejo Nacional de Investigaciones Científicas y Técnicas), 47 & 115, B1900AVW, La Plata, Argentina; dlufrano@biol.unlp.edu.ar

† These authors contributed equally to this work.

‡ These authors contributed equally to this work.

* Correspondence: cristina.marchini@unicam.it (C.M.); maurizio.falconi@unicam.it (M.F.)

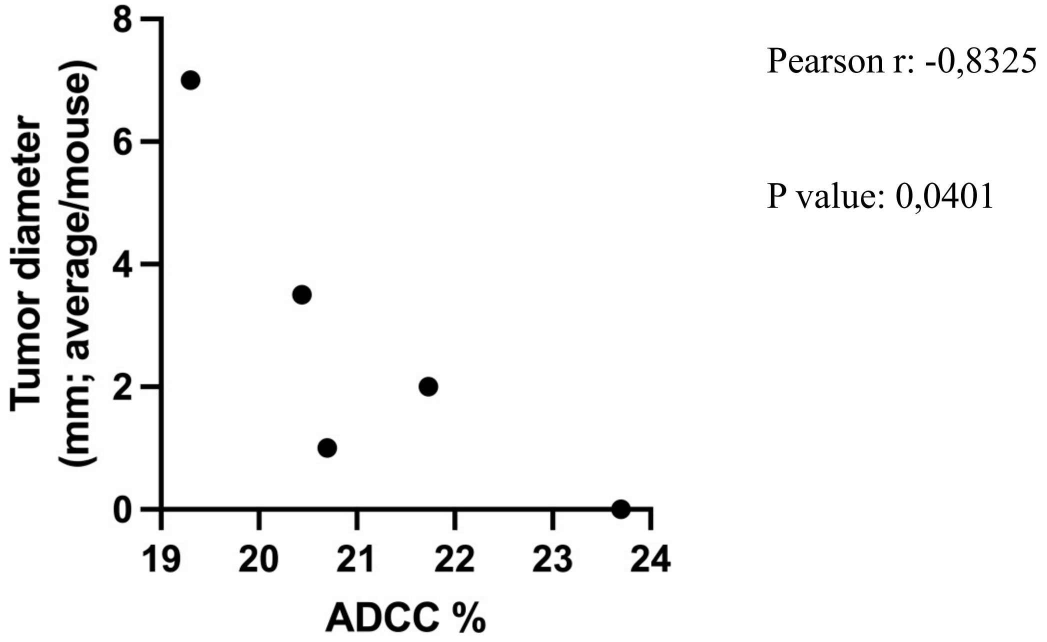

Figure S1. The percentage of ADCC and the inhibition of tumor growth are significantly correlated in individual mice. ADCC activity (%), obtained at the 100:1 E:T ratio, inversely correlated with the average of tumor diameter/mouse in ECTM-phage vaccinated mice at 24 weeks of age. The Pearson correlation coefficient (r) and the level of significance (p) of the relationship between the ADCC % and the tumor diameters are shown.

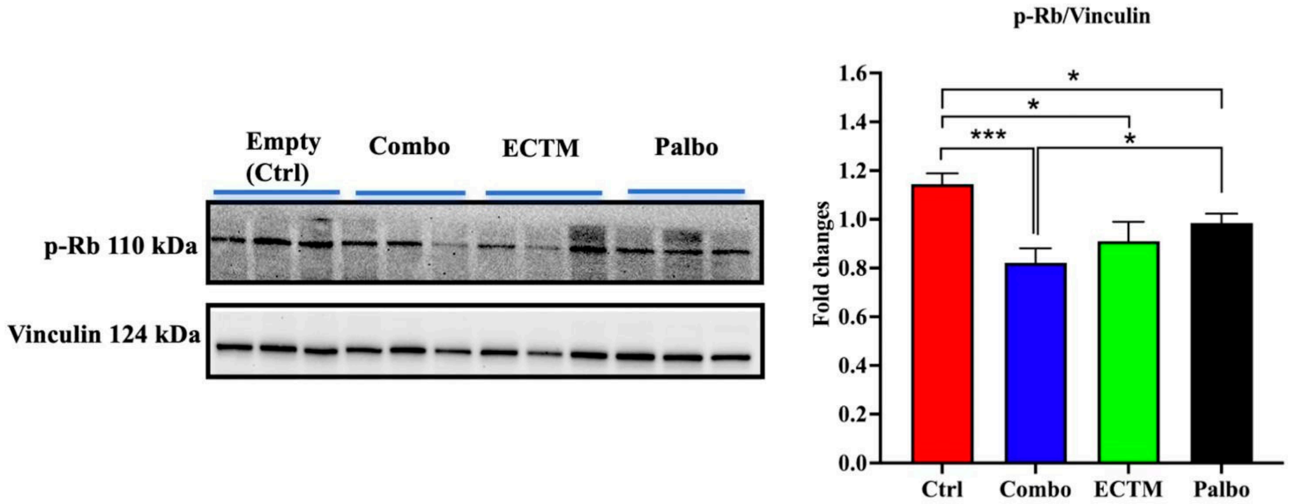

Figure S2. Left panel: Representative western blot analysis of phospho (p)-Rb, and vinculin (loading control) in tumors explanted from 30-week-old Δ16HER2 mice receiving the indicated treatments. Tumor lysates from mice receiving empty phages were used as control. Equal amounts of protein (20 µg) were loaded (n=3 mice/group). Right Panel: Densitometric quantification of pRb expression normalized with vinculin from two independent experiments. Data are expressed as mean ± SEM. Unpaired t test (* $p \leq 0.05$; *** $p \leq 0.001$).

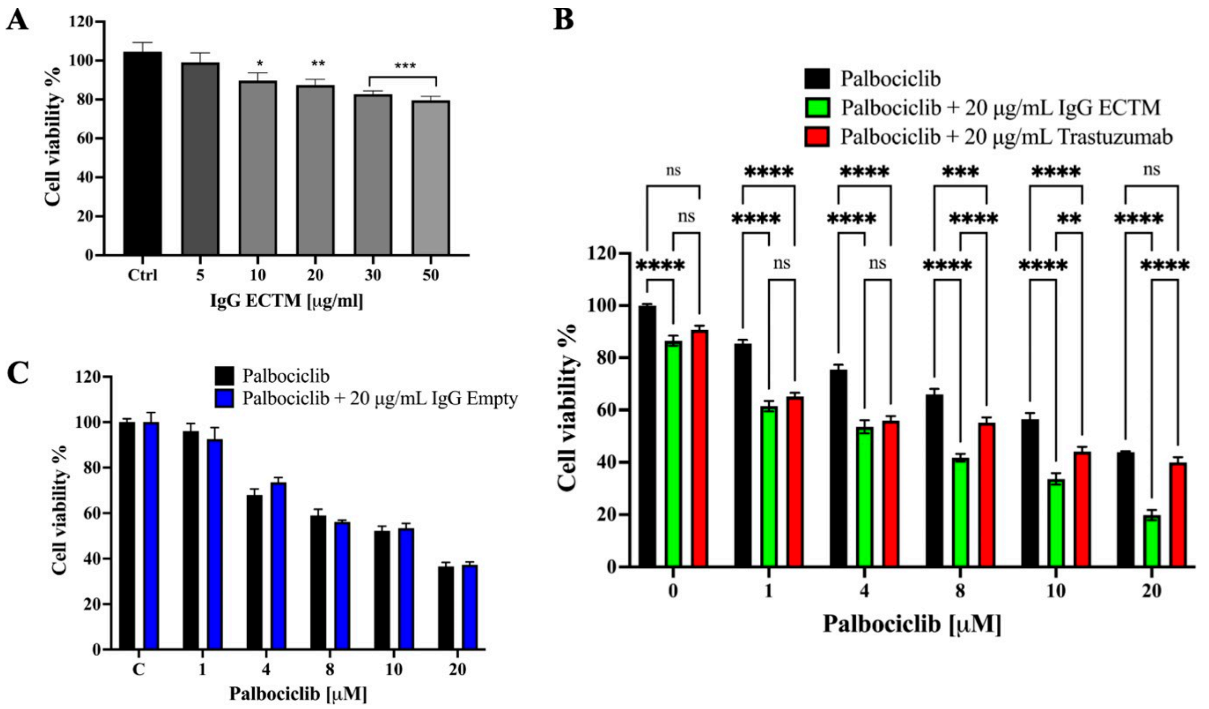

Figure S3. Anti-HER2 IgG enhanced palbociclib efficacy against HER2-positive breast cancer cells. **A**. IgG purified from ECTM-immune sera, using the Melon Gel IgG Purification Kit (Thermo Fisher Scientific, Waltham, MA, USA), decreased cell viability in SK-BR-3 cells. Cells were incubated for 72 hours in the presence of increasing concentrations of ECTM-IgG and cell viability was determined by MTT assay. The results are expressed as percentage of living cells with respect to control (untreated cells). Each bar represents the mean ±SEM (n=6). One-way ANOVA followed by Dunnett's post-hoc tests (* $p < 0.05$; ** $p < 0.01$; *** $p < 0.001$ *vs* control). **B**. Anti-HER2 IgG acted synergistically with palbociclib. SK-BR-3 cells were plated onto 96-well plates, treated with increasing concentrations of palbociclib alone (black) or in combination with 20 µg/mL anti-HER2 IgG (green) or 20 µg/mL trastuzumab (red) for 72 hours; cell viability was determined by MTT assay (n=6). Bars: means ±SEM. ** $p< 0.01$, *** $p < 0.001$, **** $p < 0.0001$. ns: not significant. One-way ANOVA followed by Dunnett's multiple comparison test. **C**. Control empty IgG, purified from sera of mice treated with empty phages, did not change palbociclib action. SK-BR-3 cells were plated onto 96-well plates, treated with increasing concentrations of palbociclib alone (black) or in combination with 20 µg/mL IgG Empty (blu) for 72 hours; cell viability was determined by MTT assay (n=6). Bars: means ±SEM.

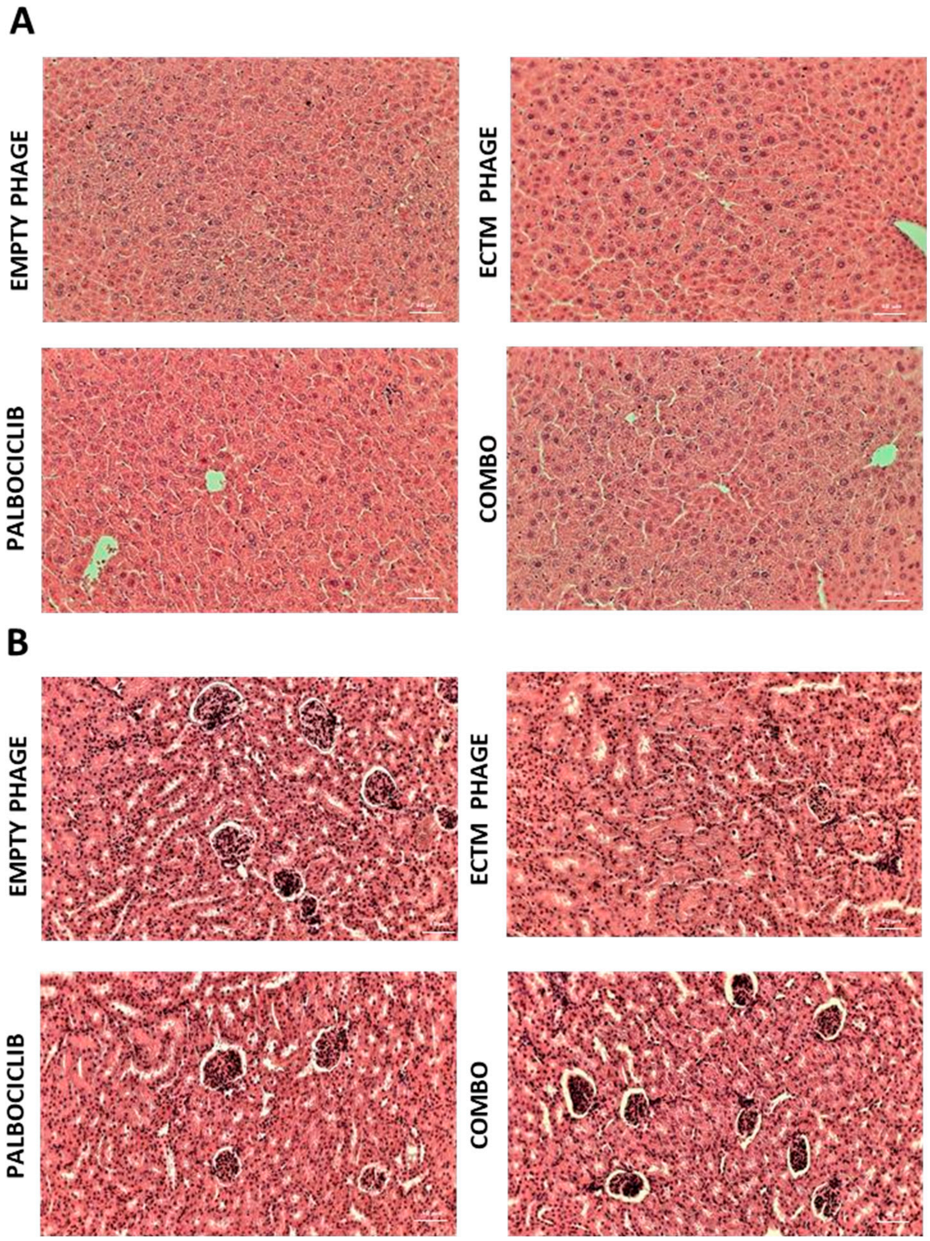

Figure S4. Morphology of peripheral organs. Representative images of Haematoxylin and Eosin histochemistry of liver (**A**) and kidney (**B**) from Δ16HER2 mice receiving empty phage (control), ECTM phage-vaccine, palbociclib, or combinatorial therapy (combo). Scale bar 50 µm; original magnification ×20.

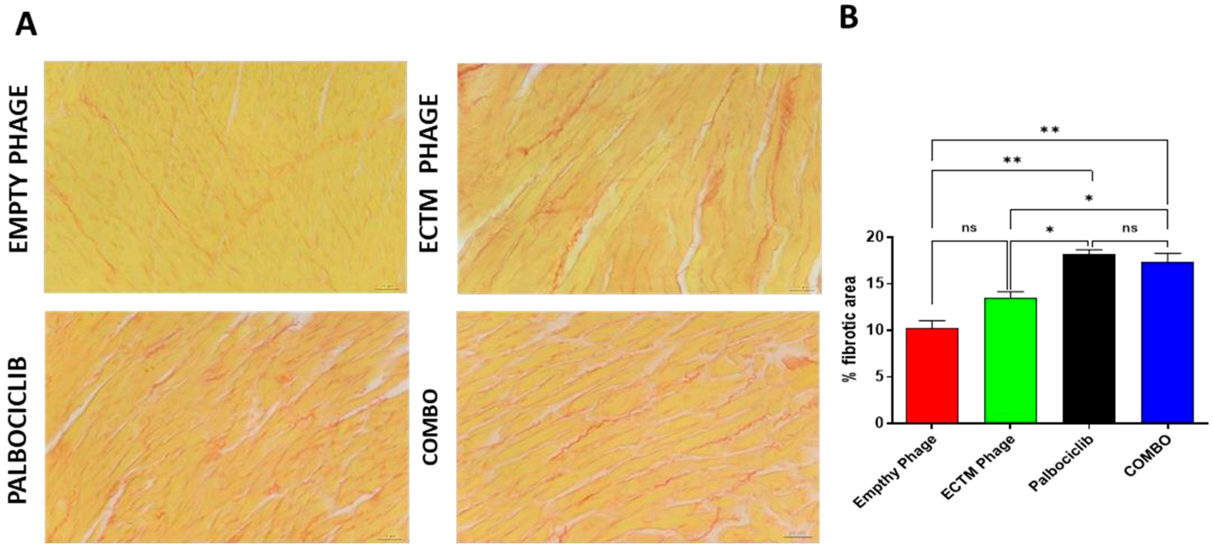

Figure S5. Evaluation of cardiac fibrosis. Representative images of Picro-Serius red histochemistry for collagen fibers (**A**) and quantification of % of fibrotic area (**B**) in heart from Δ16HER2 mice receiving empty phage (control), ECTM phage-vaccine, palbociclib, or combination therapy (combo). Scale bar 25 µm; original magnification, ×40. Data are expressed as mean ± SEM and analyzed using a One-way ANOVA test followed by Tukey's multiple-sample comparison test. ** $p < 0.01$ palbociclib or combo *vs* empty phage (control); * $p < 0.05$ palbociclib or combo *vs* ECTM phage. ns: not significant.
